# Quality improvement in exenteration for advanced pelvic malignancy: the role of a registry

**DOI:** 10.3389/fsurg.2026.1737621

**Published:** 2026-02-26

**Authors:** Tamara Glyn, Greg Turner, Frank Frizelle

**Affiliations:** 1 Department of Surgery and Critical Care, University of Otago Christchurch, Christchurch, New Zealand; 2 Department of Surgery, Te Whatu Ora Waitaha, Christchurch, New Zealand

**Keywords:** locally advanced rectal cancer, pelvic exenteration, quality improvement in surgery, recurrent rectal cancer, registries in surgery

## Abstract

Pelvic exenteration, once regarded as an ‘extreme option for hopeless cases’, has evolved into a standard of care for selected patients with advanced or recurrent pelvic malignancy. Parallel to technical and peri-operative advances, there has been a global shift towards structured quality improvement and registry-based outcome measurement. This paper outlines the historical evolution of exenterative surgery, the development of surgical quality registries, and their role in benchmarking performance. It highlights the success of international collaboratives such as PelvEx in standardising definitions and outcomes, and proposes a core dataset of key performance indicators (KPIs) relevant to exenterative surgery. These include oncological, peri-operative, and survivorship outcomes, supported by appropriate risk stratification. Establishing high-quality, prospectively maintained registries enables meaningful comparison between units, facilitates clinical governance, and strengthens advocacy for resources. Ultimately, registry-driven data are essential to refining surgical quality, optimising patient selection, and improving long-term survivorship in this complex patient cohort. This is particularly relevant with the variations in neo-adjuvant therapies.

## Introduction

Pelvic exenteration for advanced and recurrent pelvic malignancy has historically been considered to be pushing the boundaries of surgical practice (1). In contemporary practice, with advances across all aspects of this field, exenteration is now standard of care for certain patterns of disease. Where in the past, it has been the remit of isolated centres, trialling solutions to various problems and collaborating on an *ad hoc* basis, there is now an international group working together to standardise practice and optimise outcomes (2).

In parallel to the evolution of surgical practice, the landscape of quality improvement in surgery, electronic data collection methods, and the increasing importance of ’survivorship’ in complex surgery, has also progressed (3).

The most common indications for exenteration are locally advanced and recurrent rectal cancer. The treatment for this has advanced dramatically over the past five years and remains controversial. Organ preservation and the need to improve survival continues to drive intensification of neoadjuvant therapies. This has changed the landscape of local recurrence but also the degree of fibrosis and complexity in primary disease with prolonged wait times after radiation. There is significant heterogeneity in implementation of these neoadjuvant strategies and the impact on exenterative practice is yet to be fully characterised.

This paper aims to outline the importance of registries to facilitate accurate and relevant data capture, identify key performance indicators (KPIs), and suggest ways in which to incorporate this into clinical workflow in a sustainable way.

## Evolution of exenterative practice

The American gynaecological surgeon, Alexander Brunschwig, described ‘complete excision of the pelvic viscera’ in 1948 as palliation for gynaecologic and advanced rectosigmoid sigmoid colonic malignancy. The reported mortality rate for this case series of 22 patients was 25% (4). The high morbidity and mortality associated with the procedure, along with the limited perceived benefit, meant that it was not widely practiced for the better part of the remaining century. Isolated groups continued to publish their case series with modifications to urological or soft tissue reconstruction, and the inclusion of bony resection (5–7).

With advances in anaesthesia, transfusion medicine, nutrition and critical care, the peri-operative mortality improved to consistently less than 10% at the close of the 20th century (8, 9). The indications for exenteration had expanded by this point to include not only the gynaecologic malignancies, but also to primary and recurrent colorectal, urological, and sarcomatous malignancies, as well as benign indications (10). The importance of a histologically clear resection margin (R0) for both short and long-term outcomes in malignancy was appreciated, and with this, the critical role of pre-operative imaging in the planning of these operations (11). Neo-adjuvant therapy for each of the cancer streams changed and improved, increasing the likelihood of an R0 and allowing for more accurate patient selection (12).

The number of centres performing exenterative surgery increased as the mortality and morbidity profile improved. Initially, this was supported by numerous case series from multiple single centres and locally collaborating groups, however, each of these included heterogenous populations, disease processes and outcome measures, making it difficult to compare between studies (13–15).

In the last decade, the PelvEX Collaborative has been established ‘to analyse prospectively on a large scale the results of patients undergoing pelvic exenteration so as to help define guidelines to optimise patient protocols and treatment strategies’. Over 180 units contribute data to this collaborative and numerous publications have improved the understanding of contemporary exenterative practice (2, 16–18).

The nature of the patients we see for exenteration has also changed over the last 30 years. Prior to the standardisation of total mesorectal excision (TME) (39) surgery for rectal cancer and the introduction of radiation and later, neo-adjuvant chemoradiation, local recurrence was more common but also more centrally-based (19). Subsequent to these changes, an increase in lateral and bony recurrences, as well as prolonged periods from index radiation added to the complexity and morbidity of these cases, driving advances in technical and peri-operative strategies (20). With the advent of total neo-adjuvant therapy (TNT), particularly short course strategies, we have again seen a change in the nature and incidence of local recurrence (21) and, in parallel with increased organ preservation (and regrowth after radiation), contemporary practice of exenteration continues to evolve.

As exenteration has become a standard of care, the focus has shifted from simply demonstrating peri-operative safety and oncological benefit to a more holistic assessment that emphasises minimising treatment impact and optimising survivorship. This necessitates evolution in measurement tools and in the mechanisms of data capture and analysis.

## Quality improvement in colorectal surgery and exenteration

In parallel to the development of the technical aspects of pelvic exenteration, quality improvement in colorectal surgery has emerged as a key component of responsible practice. The need to measure peri-operative morbidity and mortality and quantify risk became clear in the 1980s and 1990s. The National Veterans Affairs Surgical Risk Study (NVASRS) was launched in 1991 across 44 Veterans’ Affairs (VA) medical centres in the United States (22). This study aimed to report comparative risk-adjusted peri-operative mortality rates for the involved centres. From this was born the National Surgical Quality Improvement Program (NSQIP), a prospective registry using trained nurses to capture demographic, risk factor data, operative details and 30-day morbidity and mortality. This was initially limited to the VA medical centre network, however it was later expanded beyond the VA as part of the American College of Surgeons NSQIP (ACS-NSQIP) (23). One of the strengths of this registry is in the high quality of consistent data obtained by trained, dedicated data extractors. Enrolment in the program has been shown to improve peri-operative outcomes and reduce hospital costs (24).

Quality improvement in exenteration surgery has become an important goal over the last decade as the focus has shifted from a ‘unit-based’ focus to a broader international one. There are challenges to implementing standard approaches specific to exenteration: such as the ongoing practice of exenteration cases at low volume centres, the heterogeneity of patients, pathology and terminology and the changing landscape of neo-adjuvant and adjuvant therapy. International collaboration, through PelvEx and other initiatives, has advocated for the centralisation of care and proposed minimum standards creating an environment where registries can be established (2). Burns et al (25) have proposed a standardised terminology to enable a more consistent comparison and measurement of outcomes. More recently, there has been a shift from ‘minimum standards’ to ‘best achievable standards’ with benchmarking moving the goal to overall improvement in the standard of care across the collaboration rather than focussing on ‘under-performing centres’ (26).

## Registries and their impact on outcomes

A patient registry may be defined as a ‘prospective, systematic collection of data on procedures, complications and outcomes in a defined surgical population, designed to allow benchmarking and quality improvement.’ (27) There is a long-standing use of registries in colorectal cancer. The Japan Colorectal Surgery Registry was the first to be established in 1974, collecting clinical and pathological data on colon cancer (28). Norway (29) and Sweden (30) led the way in Europe in 1993 and 1996 respectively, followed by the United Kingdom with the National Bowel Cancer Audit (NBOCA) in 2001 and the Australian and New Zealand Bowel Cancer Audit (BCCA) in 2007 (31), among others. Data from these registries have provided insights into delivery of care, for example supporting the centralisation of complex or uncommon, high risk procedures to high volume and appropriately resourced centres (32). They also highlight variations from expected outcomes and disparities in outcomes and risk factors across regions and populations.

The question of whether these registries actually have an impact on outcomes was addressed in a systematic review in 2017. Of the 17 studies included, they found evidence of ‘positive impact’ in 16. These studies were predominantly observational studies of ‘pre-‘ and ‘post-‘ implementation, with only two randomised controlled studies. They used a range of tools for measurement of impact and there was a heterogeneity in study design limiting their conclusions.

The National emergency laparotomy audit (NELA) is an example of a national, clinical quality registry which has been used to improve outcomes for patients undergoing emergency surgery in the United Kingdom. It was established in 2014 following evidence of high post-operative mortality in emergency laparotomy and initial reports following its institution demonstrated a reduction in post-operative 30-day mortality from 11.8% in 2013 to 9.5% in 2018. (NELA Project Team. Fourth Patient Report of the National Emergency Laparotomy Audit RCoA London, 2018).

This audit provides ‘real-time’, accessible, risk-adjusted data to hospitals allowing identification of outliers and both individual and system-based feedback. It has formalised the ‘risk-assessment’ aspect of the clinical flow, facilitating patient-centred discussion with the patient and their families. It can be used to interface between clinicians and administration or funding bodies, enabling more effective advocacy for resources.

Interestingly, there has been a relative plateau in the gains of some measurements over the more recent years, illustrating that act of measurement is not sufficient for improvement but rather a minimum requirement in a broader quality improvement strategy (33).

## What is required for an effective registry?

It is important to distinguish between registries initiated to improve clinical quality and those setup for administrative, billing, research or ‘device-only’ purposes.

Gliklich et al. in ‘Registries for Evaluating Patient Outcomes: A User's Guide’ delineates the key components of an effective clinical quality registry. These include a clearly defined purpose with standardised data collection of meaningful key performance indicators which allow for appropriate risk adjustment. The data quality needs to be maintained and feed into clinically implementable change. This process requires resource and governance (34).

From the planning and design stage, the importance of establishing the purpose of the registry and ensuring the design is fit for this purpose is stressed. Involvement of appropriate governance team members and stakeholder representation from the outset is necessary. Scientific content, ethics, safety, data protection and administrative processes should be considered, and the intended lifespan of the registry should be defined.

Selecting the KPIs to be included is a critical step and requires stakeholder input to ensure appropriate prioritisation. A shared, standardisable vocabulary is essential, particularly when a registry spans international health systems with geographical variation in practice. Core outcome sets may be used; however, practicality of data collection must be considered. Data collected prospectively by dedicated, trained staff are generally more accurate than secondary data extraction, although this approach is resource-dependent unless integrated into clinical workflow. Systems for evaluating data quality and auditing registry performance must be considered and incorporated into the design.

Informed consent may not be required for ‘quality improvement’ purposes, however this may then prevent the use of the data for later publication. Issues around ethics, informed consent and governance may vary between countries and health systems and should be addressed explicitly.

## Datapoints and key performance indicators in exenterative surgery

A key performance indicator (KPI) is ‘a consistently quantifiable measure of quality’. The data collected for an effective registry should include sufficient information to make appropriate risk adjustment, as well as capturing essential oncologic and survivorship outcomes. One of the challenges in standardising quality in this cohort is the heterogeneity of the patients, pathology and operations. A systematic review of outcome measures used in exenteration surgery found 1,157 unique measures in 156 individual studies; the majority of outcomes were not defined. Arguably the most important outcome measure ‘resection margin’ was variably defined between studies (35). Similar studies by the PelvEx collaborative have supported these findings in both rectal and non-rectal cancers and form the basis for current work on core outcome sets (36, 37).

Brown et al (38) have published a list of priority outcomes after exenteration for rectal cancer. They generated a ‘long-list’ of outcomes after systematic review and patient/carer interviews; a multi-disciplinary team then set up a Delphi process that involved 278 participants including clinicians, patients and carers. From the original 70 outcomes, nine priority outcomes met consensus including overall survival, disease-free survival, local recurrence-free survival, distant recurrence, psychological function, physical function, global quality of life, mobility and resection margin. This is particularly valuable work as it includes a range of clinicians as well as patients, however a more extensive dataset is probably required in a registry setting to fully capture the complexity of this patient group.

One of the key components to interpretation is appropriate risk stratification, therefore in addition to KPIs, demographic, pathologic and operative datapoints will also be needed to give context to the outcomes.

## Discussion

Pelvic exenteration is now standard of care for the management of non-metastatic locally advanced and recurrent pelvic malignancy. However, this is challenging and high risk surgery that requires infrastructure and resource to deliver. Strategies to ensure the provision of high-quality surgical care must begin with a standardised approach to data capture in order to support both developing and established units. There are pitfalls in oversimplifying this process; a systematic yet nuanced approach is necessary to enable meaningful interpretation of outcomes in this heterogeneous population. This paper presents a summary of the parallel evolution of exenterative surgery and clinical registries in surgical practice and proposes key data points necessary for an effective registry.

At a minimum, individual units should be maintaining their own prospective database of cases referred, treated and their associated outcomes. To maximise the benefit from this process, a core dataset needs to be standardised across units internationally. This is currently one of the projects being undertaken by the PelvEx Collaborative group. Once established, it will facilitate a common language allowing a better understanding of what each centre should be aiming for. This allows identification of areas for development and with appropriate strategies, an improvement in the quality of care.

Registry data may be used to advocate for appropriate resource for the service and provides accountability to funding bodies through transparent measurement of accepted outcomes. One of the key elements to a successful registry is the provision of good quality data input through trained and dedicated staff; therefore the registry itself needs to be appropriately resourced and supported.

Certain KPIs are well established, easy to measure and relatively non-controversial and will provide an obvious starting point for the dataset, however it is essential to go further than this. Patients are now consistently achieving reasonable long-term survival, making quality of life and survivorship core to the success of these operations. In particular, as we are seeing younger patients with the rise of early onset colorectal cancers, these issues will be even more relevant and complex.

## Conclusion

Where pelvic exenteration was initially consider the remit of the desperate and courageous, it is now offered as the ‘gold standard’ treatment for patients fit enough, with resectable disease. New technology, including minimally invasive access techniques and intra-operative radiotherapy are available and safe for appropriately selected patients and clinical environments, however, there is little tolerance for compromising safety. In the modern era, the fight is for incremental gains in quality of surgery and outcomes extending beyond oncological control to encompass survivorship.

In this context, each case brings the potential for deeper understanding and improving outcomes. There is a need for a registry that accurately captures the appropriate key performance indicators and the associated outcomes. Such a registry will more effectively inform decision making, risk optimisation, resource utilisation and technical improvement more effectively.

## Data Availability

The original contributions presented in the study are included in the article/Supplementary Material, further inquiries can be directed to the corresponding author.
